# Squamous Cell Carcinoma of Kidney and Its Prognosis: A Case Report and Review of the Literature

**DOI:** 10.1155/2015/469327

**Published:** 2015-01-29

**Authors:** Tapan Kumar Sahoo, Saroj Kumar Das, Chandraprava Mishra, Ipsita Dhal, Rohani Nayak, Iftekhar Ali, Debashis Panda, Saroj Kumar Das Majumdar, Dillip Kumar Parida

**Affiliations:** ^1^Department of Radiation Oncology, All India Institute of Medical Sciences College, Bhubaneswar, Odisha 751019, India; ^2^Department of Pathology, Sriram Chandra Bhanja Medical College, Cuttack, Odisha 753007, India; ^3^Department of Gynaecology, Sriram Chandra Bhanja Medical College, Cuttack, Odisha 753007, India; ^4^Department of Radiation Oncology, Acharya Harihar Regional Cancer Centre, Cuttack, Odisha 753007, India

## Abstract

Primary squamous cell carcinoma of the renal parenchyma is an extremely rare entity. The diagnosis of squamous cell carcinoma of the renal pelvis is usually unsuspected due to the rarity and inconclusive clinical and radiological features. Most of the patients are diagnosed at an advanced stage and are with poor outcome. Radical nephrectomy is the mainstay of the treatment. We reported a case of squamous cell carcinoma of the kidney in a 50-year-old female who presented with the right sided abdomen pain. The patient was treated with radical nephrectomy.

## 1. Introduction

Primary squamous cell carcinoma (SCC) of the renal pelvis is an extremely rare entity representing only 0.5% to 15% of all urothelial malignancies. It is clinically unsuspected due to its rarity and inconclusive clinical and radiological features [1]. Review of literature shows that only two cases of primary SCC of kidney have been reported to date [2]. Etiological factors are renal calculi, infection, endogenous and exogenous chemicals, hormonal imbalance, and vitamin A deficiency, although it occurs without any etiological factors [3]. We report a case of primary SCC of renal parenchyma in a 50-year-old female.

## 2. Case Report

**Figure 1:**
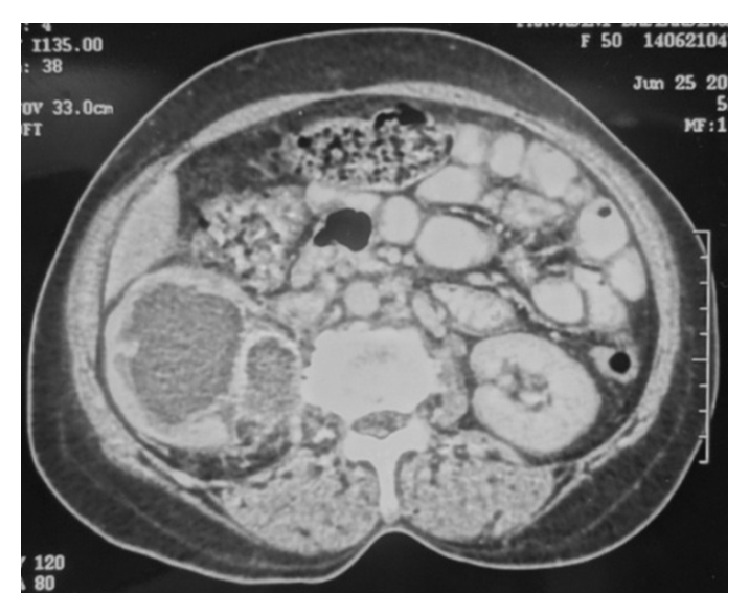
Presence of a hypodense renal mass of size approximately 6 × 8 cms in upper pole of right kidney with mild to moderate enhancement in nephritic phase.

**Figure 2:**
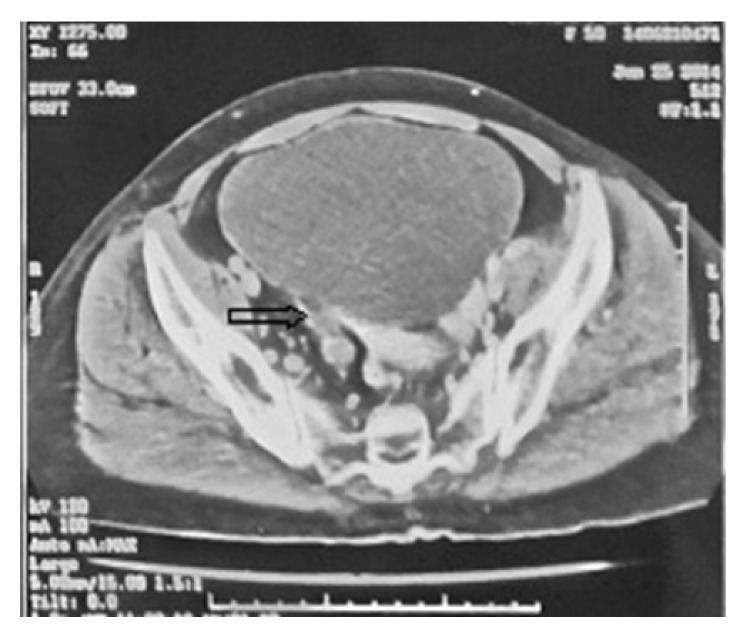
Presence of dilated ureter up to terminal part.

**Figure 3:**
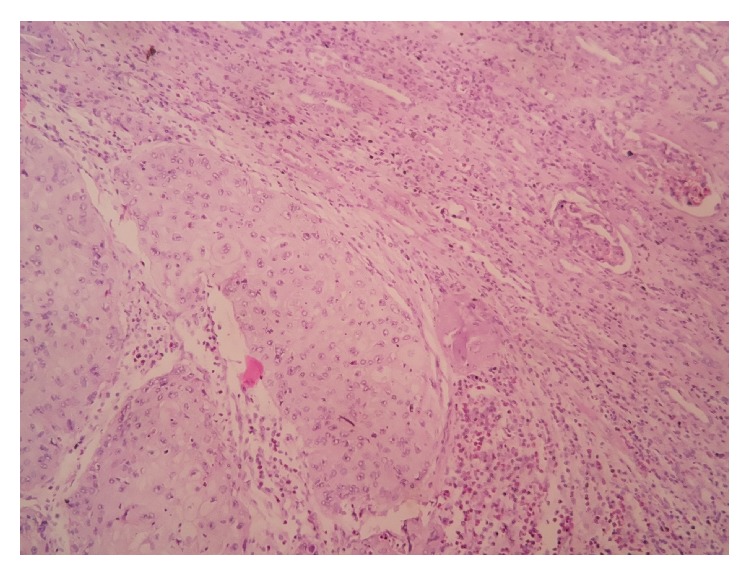
Normal glomeruli adjacent to focus of squamous epithelial carcinoma.

**Figure 4:**
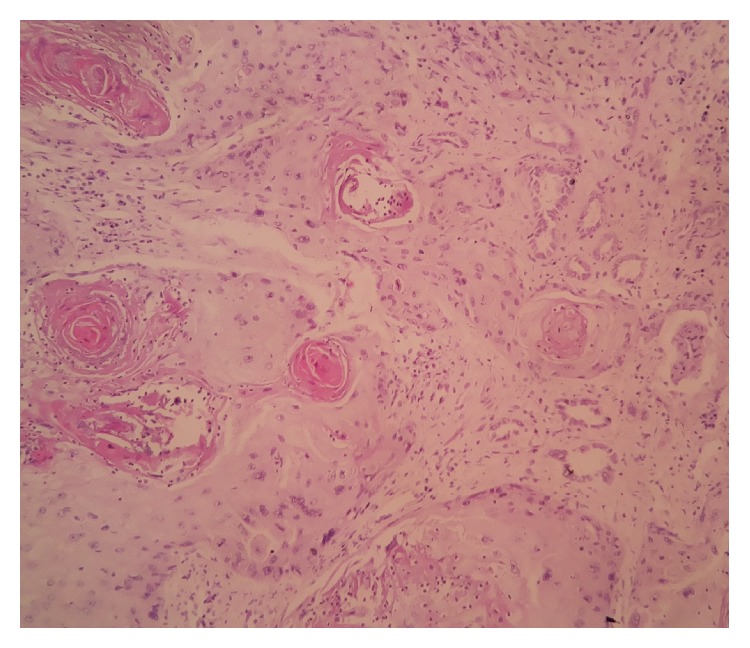
Normal tubular structures entrapped within the squamous cell carcinoma focus showing characteristic squamous pearls.

A 50-year-old female presented with pain on the right side of the abdomen since 6 months. There was no history of renal calculi, urinary tract infection, or pyelonephritis. Ultrasound examination revealed a hypoechoic mass in the upper pole of the right kidney and the right side mild hydroureteronephrosis. Contrast enhanced CT scan of abdomen and pelvis revealed presence of a mild to moderate enhancing mass of size approximately 6 × 8 cm at upper pole of the right kidney (Figure 1) and the right sided dilated ureter (Figure 2). Careful imaging study ruled out the presence of other systemic involvement. Radical nephrectomy was performed and histopathological examination revealed presence of normal looking glomeruli and renal tubules (Figure 3) along with squamous carcinomatous component and keratin pearls (Figure 4), confirming diagnosis of SCC of kidney. Renal capsule was involved but perinephric adipose tissue and hilar lymph nodes were not involved. Histopathological examination of renal pelvis was normal. Now, the patient is on regular follow-up since 6 months without any evidence of disease.

## 3. Discussion

Clear cell carcinoma is the most common type of renal malignancy followed by papillary carcinoma and chromophobe carcinoma [4]. SCC in the kidney is very unusual and is known to arise from collecting system [5]. Usually, renal squamous cell carcinoma is aggressive with high grade at the time of presentation. The most common histological type in the renal pelvis is transitional cell carcinoma followed by SCC of the kidney which is a very unusual entity. Most of them are originated from SCC of the renal pelvis. SCC consists of 0.7 to 7% of all urothelial tumours [6]. There is a female predominance and the most common age group of presentation is 50–70 years [3]. For primary renal parenchymal SCC, renal pelvis should be histologically normal. Histopathologically, squamous components in SCC of kidney are similar to other SCCs and consist of features of keratin pearls, intercellular bridges, and keratotic cellular debris. If the urothelial dysplastic element is identified along with urothelial carcinoma in situ, the tumour should be classified as primary urothelial carcinoma with squamous differentiation. Conspicuous presence of adjacent flattened urothelial keratinized squamous metaplasia in association with dysplasia supports the diagnosis of primary SCC of the renal pelvis which is rare. Such type of dysplastic or metaplastic and/or dysplastic squamous lining of epithelium was not found in our case. Squamous metaplasia of urothelium with chronic irritation is thought to cause SCC of the renal pelvis [7]. But the mechanism of SCC of the renal parenchyma is unknown, although the most probable mechanism may be similar. The present case also showed mild hydroureteronephrosis due to dilated ureter which may or may not be a risk factor but needs further study. Metastatic SCC of the kidney should be ruled out by the combination of clinical history, imaging, and histopathology [8]. In order to label it as primary SCC of renal parenchyma, metastasis from other sources should be ruled out and histopathology of the renal pelvis should be normal despite other findings. There may be possibility of an occult primary SCC somewhere in the body. In the present case, etiological factors were absent. Contrast enhanced CT scan of abdomen and pelvis revealed a solitary renal mass without any obvious other sites of lesion. Histopathological examination of pelvicalyceal area ruled out any metaplastic or dysplastic element or dysplastic squamous lining conforming the diagnosis of primary renal parenchymal SCC. Although prognosis of SCC is the same in stage-wise as urothelial cancers, they tend to occur in advanced stages [9]. Surgery is the mainstay of treatment in SCC of renal pelvis. Adjuvant treatments have marginal benefit. There is no standard guideline regarding treatment of primary renal parenchymal SCC. The present case was treated with radical nephrectomy. There is possibility of anti-EGFR therapy in renal SCC with receptor positive cases, but it needs a large number of case studies. According to previous data, the incidence, etiopathogenesis, clinical course, management, and prognosis of primary renal parenchymal SCC is inconclusive [2]. It needs a large number of case studies with proper evaluation and management.

Considering rarity of the disease, careful history taking may give a clue about the risk factors for SCC of kidney. It needs more case studies and pathological studies to establish the risk factors for primary SCC of the kidney. Proper imaging and histopathological evaluation should be done to rule out metastatic SCC of kidney and presence of dysplastic and metaplastic differentiation to study prognostic significance of such tumour. Due to extreme rarity of the tumour, there is no standard guideline for treatment. But radical nephrectomy with lymph node dissection should be the initial treatment even in metastatic tumours. Anti-EGFR therapy may be tried in EGFR positive cases.
